# Evaluation of Oral Health Status Based on DMF Index in Adults Aged 40-70 Years: Findings from Persian Kharameh Cohort Study in Iran

**DOI:** 10.30476/DENTJODS.2020.88726.1352

**Published:** 2021-09

**Authors:** Masomeh Ghoddusi Johari, Leila Moftakhar, Salar Rahimikazerooni, Ramin Rezaeianzadeh, Seyed Vahid Hosseini, Abbas Rezaianzadeh

**Affiliations:** 1 Breast Disease Research Center, Shiraz University of Medical Science, Shiraz, Iran; 2 Student Research Committee, Shiraz University of Medical Sciences, Shiraz, Iran; 3 Colorectal Research Center, Shiraz University of Medical Science, Shiraz, Iran; 4 Bachelor of Molecular and Cell Biology, Shiraz, Iran; 5 Colorectal Research Center, Shiraz University of Medical Science, Shiraz, Iran

**Keywords:** Oral health status, DMF index, Risk factors

## Abstract

**Statement of the Problem:**

Oral health is one of the most important public health problems. The DMF index is used to assess oral health status.

**Purpose:**

This study was performed to evaluate oral health status based on DMF index in adults in Fars province.

**Materials and Method:**

This cross-sectional study was performed on 8911 people aged 40 to 70 years under the Kharameh cohort study in 2020. Demographic and oral health factors were collected during interviews and clinical evaluation. T test, ANOVA, and linear regression tests were used for data analysis.

**Results:**

The mean and standard deviation of DMF index was 18.06±8.7in all individuals under study. Multiple linear regression results showed that diabetes (OR=1.1 95%CI: 0.9-1.9 *p*= 0.0001), smoking (OR=4.4 95%CI: 4-4.9 *p*= 0.0001) and underweight (OR=2.1 95%CI: 1.1-3.1 *p*= 0.0001) are the factors affecting the increase in DMF index. Other factors such as high level of education, economic and social class, flossing, and living in a village have been inversely related to the DMF index.

**Conclusion:**

The results of this study are a warning about the importance of reducing dental costs to increase the level of access of people with low economic and social levels and increase health literacy in relation to oral health.

## Introduction

Oral health is a crucial component of public health [ 1 ] that is directly related to people's health [ 2 - 3 ]. It affects many personal activities such as eating, talking, social communication and quality of life, to the extent that the World Health Organization has identified oral health as one of the most prevalent and important public health problems [ 4 ], Oral health includes a wide range of oral diseases, one of which happens to be tooth decay [ 5 ]. 

Tooth decay is a major public health concern [ 6 - 7 ] affecting people of all ages, genders, races and economic and social classes [ 2 ]. According to the World Health Organization, approximately 3.5 billion people, 35.5% of the total population, have permanent tooth decay [ 8 ]. Furthermore, about 30% of people aged 65 to 74, worldwide, do not have any healthy teeth [ 4 ]. The prevalence of tooth decay is also high in Iran. The findings of a national study conducted between 2001 and 2002 in Iran showed that the average tooth decay in 18-year-olds was 3.4 and in 35-45-year-olds; it was 11 times higher than that of everyone else [ 4 ].

Tooth decay occurs when pathogenic bacteria has covered the tooth surface [ 6 ]. Although reversible in the early stages, it progresses slowly and destroys hard tooth tissue if left untreated [ 9 ]. This can even lead to tooth loss, which in turn causes malfunction and social discomfort to people affected [ 10 ]. Tooth decay is a multifactorial disease [ 7 ], which mostly depends on eating habits such as sugar intake and individual behaviors [ 3 ]. In several studies conducted around the world, various factors have been identified in the prevalence of tooth decay, including: gender [ 11 ], age [ 12 ], level of education brushing [ 4 ], smoking [ 13 ], environmental factors [ 14 ] and economic and social class [ 11 ].

The most important indicator used to assess oral health status is the DMF index [ 4 ], which is recommended by the World Health Organization to determine and compare the rate of tooth decay in a population [ 15 ]. This index shows the number of decayed, missing and filled teeth and is used to evaluate and control oral health interventions, design policies, and implement interventional programs. Most epidemiological studies in this regard focus on children [ 6 ], thus adults have received less attention [ 4 ]. Nonetheless, evaluating adults’ oral health status is essential for planning and performing appropriate interventions for the development of oral health policies. Therefore, this study aimed to evaluate the oral health status in adults residing in Kharameh city of Fars province.

## Materials and Method

The present study is a cross-sectional descriptive-analytical study conducted using baseline data from the Kharameh cohort study (Fars province-Iran) yielded from 8911 individuals aged from 40 to 70 years old in 2020. Kharameh population-based cohort study is a part of the Persian Cohort study in Iran, which aims to investigate the risk factors for non-communicable diseases in their subjects. After obtaining consent from individuals, questionnaires were administered by trained health-care staff during face-to-face interviews. All demographic information, disease status, economic and social status and lifestyle and behavioral factors were collected during the interviews, following a clinical examinations and anthropometric measurements performed for all subjects. Other details of cohort studies can be seen in other publications [ 16 ].

The information required for this study included demographic information such as age, sex, body mass index, level of education, place of residence, smoking habits, economic and social status and diabetes history. In addition, other required data included the information on oral health, including number of decayed teeth, number of missing teeth, number of filled teeth and number of times that brushing, flossing and mouthwash was employed during the day.

Moreover, the socio-economic status of households was calculated using the principle component analysis method. The resulting asset index was subsequently divided into 5 categories (poorest - poorer - medium level - richer - richest). To calculate this index, variables related to people's owned properties were used. These variables included the type of residential ownership (owned or rented), area of residential (in square meters), number of rooms, ownership of landline telephone, washing machine, dishwasher, Flat-screen TV, refrigerator, vacuum cleaner, personal computer/laptop, access to the Internet at home, access to bathroom and toilet and finally car ownership status and its price. 

To assess the oral health status of the subjects the DMF index was used. This index was calculated based on the results of examining the condition of the teeth in terms of the number of decayed, filled and missing teeth. Oral and dental examinations were performed by trained dentists on field, using a probe, mirror and cotton rolls, in order to score each participant using the DMF index. Finally, frequency and standard deviation were used to describe quantitative variables, and frequency or frequency percentage was used for qualitative variables. After examining the normality of variables, if normal, t-test was used to examine the difference between mean DMF indices between two groups, and if not normal, Mann-Whitney test was used. Furthermore, to evaluate the difference between the mean DMF indices in more than three groups, ANOVA statistical test was applied for normal data and if not normal, Cross-Wallis test was used. To detect differences in between groups, Bonferroni test has been used. Additionally, in order to identify the factors affecting the increase in DMF index, simple and multiple linear regression was applied. Significance level was considered less than 0.05 for all tests and all analyses were performed using the STATA version 13 software.

## Results

The present study was performed on 8911 individuals aged between 40 to 70 years old from the Persian Kharameh cohort study. 43.3% of the subjects were male and 56.6% were female. 46% of people were in the age group of 40 to 50 years old, 34.4%, 50 to 60 years old and 19.5%, 60 to 70 years old. 65.3% of subjects did not use a toothbrush; only 28.2% brushed once a day, 5.8% twice a day, and 2.3% of people brushed three times a day. Further demographic and oral health information of the subjects are shown in Table 1.

**Table 1:** Demographic characteristics and oral health information of adults aged 40 to 70 years old living in Kharameh city

Variable	Level	N	%
Age	40-50	4103	46
	51-60	3070	34.4
	61-70	1738	19.5
Sex	Male	3866	43.3
	Female	5045	56.6
Education	Illiterate	4372	49
	Primary	2349	26.3
	Secondary	1009	11.3
	High-school	636	7.14
	University	545	6.12
Smoking habits	No	6884	77.2
	Yes	2027	22.7
Residence	Urban	3195	35.8
	Rural	5716	64.1
BMI	Under weight	287	3.22
	Normal	3082	34.6
	Over weight	3820	42.88
	Obese	1719	19.3
SES	Poorest	1682	18.8
	Poor	1752	19.6
	Middle	1765	19.8
	Richer	1816	20.3
	Richest	1896	21.3
Diabetes History	No	7668	86.05
	Yes	1243	13.9
Mouthwash Use	No	8829	99.08
	Yes	82	0.92
Brushing Habits	Once/Day	2519	28.2
	Twice/Day	520	5.8
	Three times/Day	209	2.3
	None	5663	65.3
Flossing	No	8314	93.3
	Yes	597	6.7

Based on the results of this study, the mean and standard deviation (SD) DMF in all subjects was 18.06± 8.7 and the mean (SD) number of decayed, missing, and filled teeth was 5.9±5.6, 10.7±7.5, and 2.7±1.3 respectively. The results of independent t test and Mann-Whitney test in evaluating the difference between the mean DMF at the levels of two-state qualitative variables showed that there was a statistically significant difference in the mean DMF between women and men (*p*< 0.0001), smokers and non-smokers (*p*< 0.0001), urban and rural populations (*p*< 0.0001), as well as between subjects who floss and those who do not (*p*< 0.0001). However, at different levels of mouthwash use, no statistically significant difference was observed in DMF index numbers (Table 2).

**Table 2:** Mean number of decayed, missing, filled teeth and DMF index based on demographic variables of factors related to oral health in adults 40 to 70 years old living in Kharameh city

Variable	Level	Decayed	Missing	Filled	DMF	*p* Value
		Mean ±S.D*	Mean ±S.D*	Mean ±S.D*	Mean ±S.D*	
Age	40-50	6.1±5.7	10.3±7.3	1.4±2.8	17.9±8.7	0.3
	51-60	5.9±5.5	11.08±7.6	1.2±2.6	18.2±8.8	
	61-70	5.6±5.4	10.8±7.6	1.4 ±2.8	17.9±8.6	
Sex	Male	6.8±6.2	11.1±7.8	1.1±2.6	19.2±9.2	0.0001
	Female	5.3±5.1	10.3±7.2	1.4±2.8	17.1±8.2	
Education	Illiterate	6.4±5.6	12.2±7.6	0.5±1.7	19.2±8.6	0.0001
	Primary	5.9±5.6	9.9±7.1	1.4±2.7	17.3±8.6	
	Secondary	5.7±5.8	9.4±7.09	2.1±3.2	17.3±8.9	
	High-school	5.4±5.6	8.7±6.8	2.7±3.5	16.9±8.6	
	University	3.2±4.3	6.31±5.6	4.3±4.1	13.6±7.6	
Smoking habits	No	5.5±5.3	9.9±7.1	1.5±2.9	17.01±8.4	0.0001
	Yes	7.5±6.4	13.2±8.03	0.81±2.2	21.6±8.8	
Residence	Urban	5.7±5.7	10.5±7.08	2.4±3.6	18.7±8.3	0.0001
	Rural	6.1±5.5	10.8±7.7	0.72±1.9	17.6±8.9	
BMI	Under weight	7.8±6.5	14.5±8.9	0.4±2.5	22.7±8.9	0.0001
	Normal	6.6±5.9	11.8±7.9	1.08±2.5	19.5±8.8	
	Over weight	5.5±5.4	10.02±7.1	1.5±3.01	17.1±8.5	
	Obese	5.4±5.2	9.5±6.7	1.4±2.7	16.4±8.2	
SES	Poorest	7.07±5.8	11.8±7.8	0.2±0.9	19.4±8.8	0.0001
	Poor	6.3±5.5	11.9±7.7	0.5±1.6	18.8±8.8	
	Middle	6.1±5.6	10.9±7.5	0.08±2.09	17.9±8.7	
	Richer	5.8±5.7	10.1±7.1	1.7±2.9	17.6±8.6	
	Richest	4.6±5.3	8.8±6.8	3.1±3.8	16.7±6.3	
Diabetes History	No	5.9±5.6	10.5±7.5	1.3±2.7	17.8±8.7	0.0001
	Yes	6.3±5.9	11.5±7.5	1.3±2.7	19.2±8.4	
Mouthwash Use	No	5.9±5.6	10.7±7.1	11.3±2.7	18±8.7	0.13
	Yes	5.9±5.4	8.5±6.1	2±3.6	16.6±8	
Brushing Habits	Once/day	4.8±5	8.5±6.5	2.2±3.3	15.2±7.9	0.0001
	Twice/Day	6.4±6.1	9±6.8	1.9±3.3	17.5±8.5	
	Three times/Day	5.9±5.7	9.7±6.9	2±3.4	17.6±8.3	
	None	6.4±5.8	11.8±7.7	0.8±2.3	19.2±8.8	
Flossing	No	6±5.6	10.9±7.5	1.2±2.6	18.2±8.7	0.0001
	Yes	5±5	4.9±6.7	3.2±3.9	15.1±7.7	

* S.D: Standard Deviation

Value: T-test and Mann-Whitney for two-state qualitative variables, Anova and Kruskal-Wallis for qualitative variables with more than two-states

Based on the results of ANOVA and Kruskal-Wallis analyses, for investigating the difference in the mean DMF in the levels of qualitative variables with more than two levels, significant statistical difference between the mean DMF at different levels of education (*p*< 0.0001), body mass index (*p*< 0.0001), economic and social class (*p*< 0.000) and use of toothbrush (*p*< 0.0001) was witnessed, whereas this was not the case in between different age levels (Table 2).

The results of simple linear regression also displayed a statistically significant relationship between mean DMF and education level, smoking, place of residence, body mass index, socioeconomic status, diabetes and flossing.

Multiple linear regression test was used to control confounders. The results showed that diabetes, smoking and being underweight were effective factors in increasing the DMF index in such a way that after controlling for other variables, the mean DMF in diabetic individuals was 1.4 units higher than non-diabetics (*p*< 0.0001), smokers 4.4 units higher than non-smokers (*p*<0.0001) and in low weight people, 2.1 units more than people with normal BMI (*p*< 0.0001). 

Other factors had a protective role so that the mean DMF after controlling for other factors in people with dental flossing habits was 1.3 units less than those who did not floss (*p*< 0.0001), and in the rural population it was 2.7 units less than the urban population (*p*<0.0001). 

Furthermore, with increase in the level of education, the average DMF index decreased in such a manner that after controlling for other factors, the average DMF index were 2 units, 2.9 units, 3.4 units and 5.7 units less in subjects with primary education, secondary education, high school education and university education respectively in comparison to those with no education at all. Other factors including economic and social class are shown in Table 3.

**Table 3:** Results of simple and multiple linear regressions to predict the factors affecting the DMF index in adults aged from 40 to 70 years old in Kharameh

Variable	Level	Simple linear regression		Multiple linear regression	
		(CI%95)β	*p* Value	(CI%95)β	*p* Value
Education	Illiterate	-	-		
	Primary	-1.9(-1.5,-2.3)	0.0001	-2(-1.5,-2.4)	0.0001
	Secondary	-1.9(-1.3,-2.5)	0.0001	-2.9(-2.3,-3.5)	0.0001
	High-School	-2.3(-1.6,-3)	0.0001	-3.4(-2.7,-4.1)	0.0001
	University	-5.3(-4.5,-6.1)	0.0001	-5.7(-4.9,-6.6)	0.0001
Smoking Habits	No	-	-	-	-
	Yes	4.6(4.1,5)	0.0001	4.4(4,4.9)	0.0001
Residence	Urban	-		-	
	Rural	-1.1(-0.7,-1.4)	0.0001	-2.7 (-2.3,-3.1)	0.00001
BMI	Normal	-		-	
	Under weight	3.2(2.1,4.2)	0.0001	2.1(1.1,3.1)	0.0001
	Over weight	-2.3(-1.9,-2.7)	0.0001	-1.7 (-1.3,-2.1)	0.0001
	Obese	-3(-2.5,-3.5)	0.0001	-2.4(-1.9,-2.9)	0.0001
SES	Poorest	-	-	-	-
	Poorer	-0.36(-0.9,0.21)	0.2	-0.33(-0.8,0.2)	0.3
	Middle	-1.2(-0.63,-1.7)	0.0001	-0.95(-0.3,-1.5)	0.003
	Richer	-1.5(-0.93,-2.09)	0.0001	-1. 2(-0.5,-1.8)	0.001
	Richest	-2.4(-1.8,-3)	0.0001	-1.2(-0.5,-1.8)	0.005
Diabetes History	No	-	-	-	-
	Yes	1.3(0.8-1.8)	0.0001	1.4(0.9-1.9)	0.0001
Flossing	No	-	-	-	-
	Yes	-3.3(-2.4,-3.8)	0.0001	-1.3(-0.6,-2)	0.0001

## Discussion

In this study, the mean DMF index (standard deviation) in all subjects was 18.06±8.7. Although, no statistically significant difference between the mean DMF in the age groups of 40 to 50, 50 to 60 and 60 to 70 was observed, but, we must keep in mind that the number of people in all three age groups were not equal, and people between the ages of 61 and 70 were about half the size of any other age group. On the other hand, the younger generation is at higher risk of tooth decay due to higher consumption of sweets and less consumption of completely natural dairy products. Of course, today, due to the higher treatment and diagnostic facilities, people treat their decayed teeth faster, while in the past, these facilities were less.

These can be effective in reducing this difference. Mean DMF index in this study was higher than other previously conducted similar studies. In a previous study conducted in Iran’s Kurdistan, the DMF index mean in the age group of 35 to 45 years was equal to 7.8 [ 4 ], and in another study this number was in Iran 14.8. In Japan 12.2, Malaysia 12.1, South Korea 5.2 and Turkey 10.8 [ 17 ]. Additionally, in a study conducted in Kosovar, the mean DMF index was 11.6 for people aged 35 to 44 and 13.6 for people aged 45 to 65 [ 6 ]. In eastern China this number was 3.5 [ 18 ], in Italy 4.3 [ 11 ] and 14, between Greece’s 35-75 years old population [ 14 ]. This difference in the average DMF index could be due to cultural, economic and social differences as well as access to oral health services and healthcare in general in the study location. Another reason could be attributed to the differences existing in these populations given that they could not be completely similar.

The results of this study demonstrated that mean DMF in smokers was 4.6 units higher than non-smokers, therefore being the most effective in increasing the mean of this index compared to any other factor under study. Yet only few studies on smoking in the adult population of Iran have been carried out hence further investigation in this regard is necessary. In addition, in the present study, the mean DMF in low weight individuals was 3.2 units higher than individuals with normal weight, consistent with the results of a similar study in South Korea [ 8 ], nevertheless in a number of other studies, obesity has caused an increase in DMF index [ 3 , 18 - 19 ]. 

Higher education, economic and social levels in our study reduced the DMF index, in consistency with the results of many other prior studies [ 20 - 21 ]. Research carried out in Greece [ 14 ], Italy [ 11 ] and between 20 to 90-year-old Iranians and Brazilian adults, concluded that a higher level of education, economic and social class reduces the DMF index. However, it should be stressed that people with higher levels of education are in better economic, social and financial conditions, therefore oral health care facilities are more accessible to them, hence reducing the DMF average.

In our study, subjects living in the rural areas had a reduced DMF index mean; these results are in contrast with a number of other conducted studies including a study carried out in Vietnam reporting that the average DMF in urban areas is lower than those of the rural [ 21 ]. In the study of Kosovar adults, no difference in the DMF mean between rural and urban populations was apparent [ 6 ]. This variation in observations from different regions could be due to diverse cultures and lifestyles of subjects under study. As an example, in rural Iran, consumption of sweets that cause tooth decay happens to be less, whereas local milk and dairy consumption is higher in comparison to the urban population.

In our study, there was no difference in mean DMF between male and female subjects of different age groups. While some studies have shown mean DMF increases with older age [ 14 , 21 ] and in women [ 14 ], other studies, on the contrary, have shown that this index is higher in men [ 20 ].

Moreover, in our study among effective components on oral hygiene, flossing, like many other studies [ 22 ], reduces the mean DMF in individuals. In general, we should keep in mind that subjects in different studies do not fall in the same age groups; these studies have been conducted in different parts of the world with different cultures, economic and social conditions, insurance coverages and health care services with varied oral and tooth care procedures. Therefore, we must be cautious in comparing the results of these studies.

This study is cross-sectional and therefore the cause and effect relationship of some of the variables in the study may not be properly identified. Our study was also limited to a certain area with cultural, economic and social conditions specific to people living in that area. As a result, the results may not be easily generalized to other communities. However, this study, compared to many other studies in this field, has been performed on a large number of people.

## Conclusion

As the results of the present study show, low levels of education, economic and social status along with low weight are the factors that increase the mean DMF. This indicates little access to health information and dental care services. It should be emphasized that in Iran, most dental services are not covered by insurance services, while the cost of these services tend to be very high. As a result, people with lower income and of lower social class, face financial difficulties for benefiting from oral health services, thus their DMF index increases. The results of this study help health policy makers take appropriate measures to solve the unfavorable health situation of the oral health in adults, including covering these benefits under health insurance services.
